# The involvement of musculoskeletal system and its influence on postural stability in children and young adults with cystic fibrosis

**DOI:** 10.1186/s13052-017-0426-0

**Published:** 2017-11-21

**Authors:** Ozge Kenis-Coskun, Evrim Karadag-Saygi, Yeliz Bahar-Ozdemir, Yasemin Gokdemir, Bulent Karadag, Onder Kayhan

**Affiliations:** 1 Semsi Denizer Cad Dr Lutfi Kirdar Kartal Research and Training Hospital, Physical Medicine and Rehabilitation Department Istanbul, 34890 Istanbul, Turkey; 2 Marmara University Medical Faculty Physical Medicine and Rehabilitation Department Istanbul, Istanbul, Turkey; 3 Marmara University Medical Faculty Pediatric Pulmonology Department Istanbul, Istanbul, Turkey

**Keywords:** Cystic fibrosis, Posture, Postural stability, Musculoskeletal system, Spine

## Abstract

**Background:**

Cystic fibrosis (CF) affects the musculoskeletal system via a multifactorial pathway that includes vitamin D deficiency and involvement of respiratory muscles such as intercostals due to recurrent upper and lower respiratory tract infections. Eventual result is the deterioration of musculoskeletal health and posture in CF patients. Postural stability is directly affected by posture and can be compromised in every musculoskeletal problem. The aim of this study is to evaluate musculoskeletal system and postural stability in patients with CF.

**Methods:**

Patients with CF over six years of age and age and sex-matched control groups were included in the study. Cobb angle and thoracic kyphosis angles were measured on the spine radiographs. Both patients and control group were examined with pediatric gait, arms, legs and spine scale (pGALS). They also were evaluated with a NeuroCom Balance Master for their postural stability.

**Results:**

Fifty-one patients with CF and 94 healthy controls participated in the study. In results of the pGALS examination, CF group had significantly more pathological findings than the control group in lower extremity appearance and movement (*p* = 0.006 and *p* = 0.01) and spine appearance and movement (*p* = 0.001 and *p* = 0.022) domains. The tandem walking speed was significantly higher in controls with a mean of 24.45 ± 7.79 while it was 20.47 ± 6.95 in the CF group (*p* = 0.03). Various limits of stability parameters also showed significant differences. Medium correlations were found between musculoskeletal examination and postural stability parameters.

**Conclusion:**

In patients with CF, a systematic but simple musculoskeletal examination can detect pathologies, which are more frequent than the normal population. These pathologies show a medium correlation with the involvement of postural stability.

## Background

Cystic fibrosis (CF) is the most common autosomal recessive disorder among individuals of European ancestry. The life expectancy for CF has dramatically increased in the recent decade with advancements in medical care [1]. This increase in life expectancy has broadened the span of morbidities in CF and started involving other systems like musculoskeletal system.

The musculoskeletal involvement of CF is indirect, usually seen with deformities of the spine as the disease progresses. There is an increased incidence of scoliosis in patients with CF. It has been shown in a previous study that, among 316 CF patients the prevalence of scoliosis was 15.6%, which is 20 times higher than the prevelance of the area the research has been conducted [2]. The exact cause is not clear; it is thought to be a result of the combination of vitamin D deficiency, use of inhaled corticosteroids and the strain on postural muscles as the disease progresses [2, 3]. Thoracic kyphosis also increases due to decreased bone mineralization. These changes can lead to musculoskeletal pain, such as persistant back pain, especially in patients in adulthood. Chronic pain is yet another added burden to this patient group, and may exacerbate low mood and decrease motivation, therefore must be prevented if possible [4]. Various studies defined the spine involvement in CF [2, 5–8], but the number of studies examining the entire musculoskeletal system is limited [9].

Postural stability is the ability to keep the center of mass within the base of support in standing still and during movement which is the combined effort of sensory, motor and biomechanical systems [10–12]. The pathologies involving any of these systems end up disrupting the postural stability. There are two studies investigating this relationship in fourteen adult patients with CF [13, 14]. The exact functional implications of this spine involvement are not defined clearly, and the data showing the involvement of postural stability and its relationship to musculoskeletal health is scarce.

Using a standardized question and physical exam, we hypothesized that the detected number of musculoskeletal pathologies in patients with CF are higher than typically developing children. Also, we hypothesized that the musculoskeletal involvement affects the postural stability parameters in patients with CF.

## Methods

This study was conducted between February 2014–December 2014 in Marmara University Physical Medicine and Rehabilitation Clinic. The inclusion criteria were the presence of a diagnosis of CF according to the criteria depicted in Cystic Fibrosis Foundation 2008 consensus report [15] and being above six years of age. Exclusion criteria were a history of an acute exacerbation and/or internalization in an intensive care unit within the last three weeks, history of a lung or liver transplant; concomitant neurological, cardiovascular, metabolic, rheumatic or vestibular diseases; physical disabilities impairing locomotion; orthopedic problems and a history of musculoskeletal system operations.

**Fig. 1:**
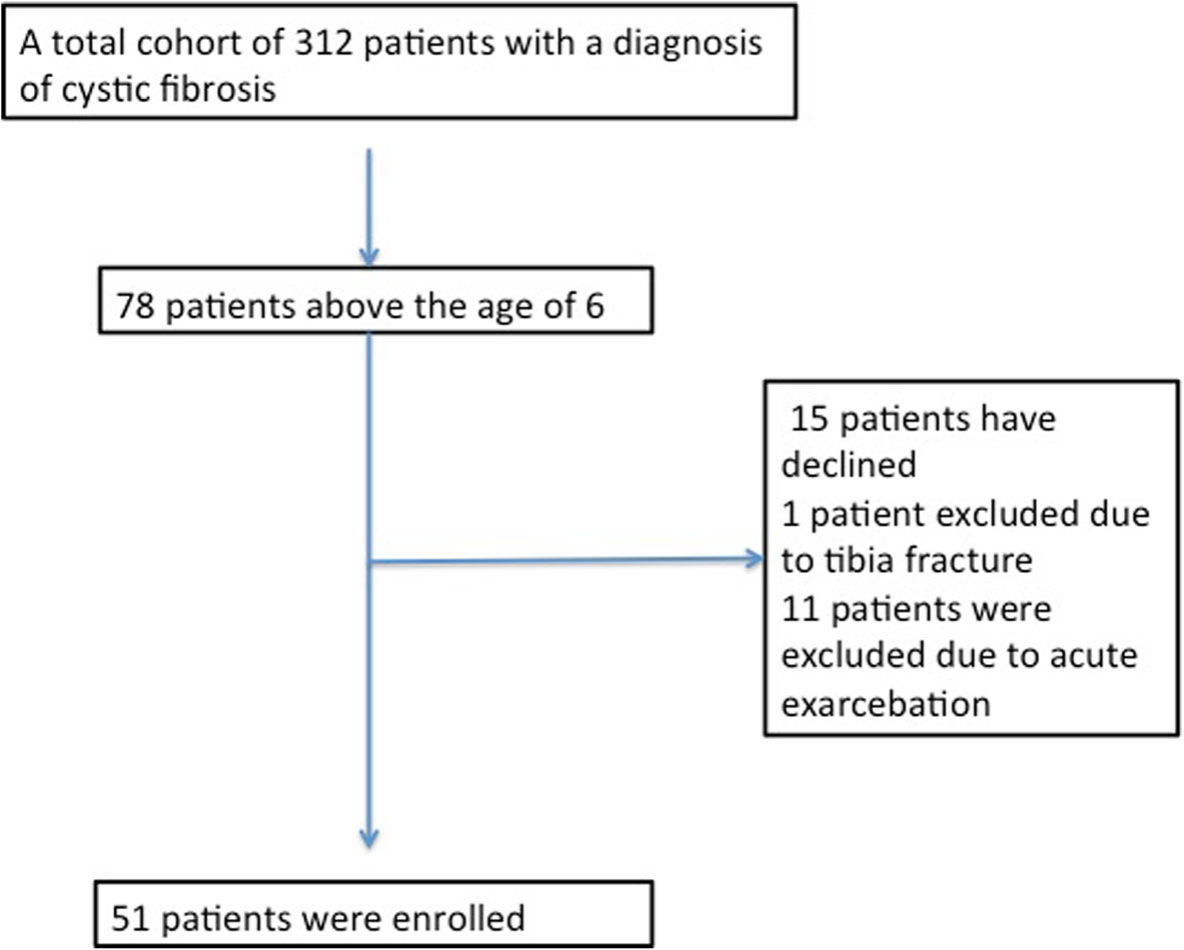
Patient enrollment process of the study

Among the cohort of 312 patients that are currently followed by the clinic 78 were above the age of six. All the patients were reached on the telephone and were informed of the research eleven patients were excluded due to their recent history of acute exarcebation, and one patient was excluded due to a recent tibia fracture. Fifty-one patients accepted to join the study. (Fig. 1) This study was carried out in accordance with the recommendations of Marmara Universtiy Ethical Committee with written informed consent from the parents for the patients under the age of 16 and the subjects above 16 years of age. All subjects gave written informed consent in accordance with the Declaration of Helsinki. The protocol was approved by the Marmara Universtiy Ethical Committee.

Age (years), height (metres), weight (kilograms), and body mass index (BMI) (kg/m2) Z-scored were recorded for the CF group. All the participants in the CF group underwent spirometry and their FEV1, FVC, FEV1/FVC, and PEF scores were recorded. All the measurements and postural stability evaluations were done on the same day. Patients were grouped according to their FEV1 levels as mild (above 70%), moderate (between 70 and 40%) and severe (below 40%) [16]. Patients with 25-OH-vitamin-D levels below 30 ng/ml were labeled as vitamin D deficient [17].

After the analyses of CF group have been finished, age and sex matched controls without a history of musculoskeletal and pulmonary pathologies who are referred to us mainly by the nearby district schools for a routine musculoskeletal examination were selected. The control group was not derived from a pool but rather every subject and their parents were asked to volunteer on a personal basis and the controls that have accepted are included in the study. Subjects in the control group underwent heigh and weight measurements, pGALS examination and postural stability assessment. They did not undergo pulmonary function tests, blood tests or received radiographs due to ethical considerations.

### Musculoskeletal examination

The participants were evaluated with pediatric Gait, Arms, Legs and Spine (pGALS) system. pGALS examination system was developed based on GALS in 2006. It is a fast and efficient way of musculoskeletal examination in school-age children [18]. The physician performing pGALS (EKS) was educated about the system. It includes three scanning questions and 17 maneuvers to examine the upper extremity, lower extremity, spine and temporomandibular joint. Pathologies were reported according to movement and appearance [19].

### Spine radiographs

The spine radiographs were taken for the CF group and were evaluated with Surgimap® (Nemaris Inc. New York, USA) system. Two blinded researchers (YBO and OKC) measured the Cobb and kyphosis angles twice [20]. Thoracic kyphosis angle was measured between the angles of the upper border of T4 and the lower border of T12 vertebrae [20]. Scoliosis was defined as a Cobb angle of more than 10 degrees [21]. Increased thoracic kyphosis were defined as a kyphosis angle above 40 degrees [22].

### Postural stability evaluations

The evaluations done with NeuroCom Balance Master® (Natus Medical, San Carlos, California, USA) are as follows:

- Modified (mCTSIB): Participants were required to stand on a firm surface with eyes open and closed for 30 s respectively. The same process was repeated on a foam surface. The sway velocity (degrees/s) in center of gravity (COG) was measured. Every measurement was done three times, and the mean of these measurements was used.
- Limits of Stability (LOS): This test required participants to transfer their COG toward eight targets spaced at 45° intervals around the body’s COG. Participants were asked to keep their bodies in a straight line and use their ankles as their movement axis to reach the targets, which are preset according to the participants’ height. Participants were asked to reach each of the 8 targets separately. Target 1 was forward, 2 was forward-right, 3 was straight right, 4 was backward right, 5 was straight backward, 6 was backward- left, 7 was straight left and 8 was forward left. Target placement takes into account the conversion of the angular motion of leaning to linear movement of the COG represented on the screen. Directional control (DC), endpoint excursion (EPE) (percentage of the distance achieved toward a target on the initial movement), and movement velocity (MVL) (average speed of COG movement based on the middle 90% of the distance, measured in degrees per second) were measured. The results of these tests were abbreviated as DC1 meaning directional control forward, and so on.
- Tandem walk: This test requires the participant to tandem walk on the bold black line in the middle of the platforms and wait until the testing ends at the end of the line in tandem position. Step width (cm), speed (cm/s) and end sway velocity (degrees/s) were measured. The mean of three measurements was used.
- Sit to Stand (STS): Participants were required to sit on a wooden box placed on the platform with their hands on the side and the legs closed. Then they were asked to stand up on cue and stand for 5 s. Tests were repeated three times. Weight transfer time (seconds), weight transfer index (percent of body weight) and sway velocity (degrees/s) were measured and the mean of three measurements were used.

### Statistical analyses

With an alpha = .05 and power = 0.80, the projected sample size has been calculated as approximately 40 subjects for cystic fibrosis group and 80 subjects for the control group for a between groups comparison of limits of stability parameters to detect a 30% difference between groups.

SPSS for Windows 16 was used for statistical analysis. Descriptive statistical methods like mean, standard deviation and median have been used. In analyzing ordinal data, means were compared by Student t test or Mann-Whitney-U test according to the distribution of the data. A level of *p* < 0.05 has been accepted as significant.

## Results

Fifty-one patients with CF and 94 healthy controls participated in the study. There were no significant differences in sex and age between groups (*p* = 0.331 and *p* = 0.8 respectively). In CF group there were 26 males and 25 females, and there were 52 males and 42 females in control group. Ages, BMI Z-scores, vitamin D levels, FEV1 and FVC values, treatment durations of the patient group are given in Table 1 and the characteristics of the control group are shown in Table 2.

**Table 1:** Demographic properties of the patients with CF

	Minimum	Maximum	Mean	Std. Deviation
Age (years)	6	28	14.04	5.344
FEV1 (%)	35	118	77.70	20.420
FVC	47	118	80.9	17.4
FEV1/FVC (%)	61	115	98.5	12.2
Vitamin D level (ng/ml)	3.00	47.15	17.86	9.56
BMI z scores	−3.30	1.46	−.69	1.18
Treatment Duration (years)	1	25	11.30	5.72

**Table 2:** Characteristics of the control group

	CF	Control	*p* value
Age mean SD (min-max)	14.04 5.34 (6–28)	12.48 4.1 (7–24)	0.80
Gender	female 25	female 42	0.61
	male 26	male 53	
BMI mean SD (min-max)	18.56 3.41 (13.01–26.51)	19.10 3.8 (13.1–25.61)	0.72

FEV1 was inversely correlated with age (*r* = −0.524, *p* = 0.001). FEV1 and FVC levels showed no correlation with Cobb angle and thoracic kyphosis angle. Cobb angles seem to increase with age (*r* = 0.326 *p* = 0.04) while there were no correlations between age and thoracic kyphosis angle. Forty-six of the patients (79.3%) had lower levels of vitamin D. Vitamin D levels had an inverse correlation with Cobb angle (*r* = 0.634 p = 0.04). BMI Z-scores did not show any correlation with vitamin D levels when it is checked as a surrogate measure for nutrition.

### Results of pGALS examination

As previously mentioned, pGALS examination consists of two major parts: Scanning questions and musculoskeletal examination. According to the results of scanning questions, there was a significant difference between groups only in answer to question ‘Do you experience difficulty going up and down the stairs?’ (Table 3).

**Table 3:** Answers to the scanning questions in pGALS

		CF group	Control group	*P* value
Are there any pain or stiffness in your joints?	Yes	15	30	0.775
	No	25	56	
Can you dress on your own?	Yes	45	86	0.634
	No	0	0	
Do you experience difficulty going up and down the stairs?	Yes	15	3	<0.0001
	No	30	78	

There were no differences between the groups in upper extremity appearance and movement examination. In all other parameters, CF group had significantly more pathological findings (Table 4).

**Table 4:** Results of pGALS examination

		CF group	Control Group	*P* value
Lower extremity- Appearance	Normal	37	76	0.006*
	Pathological	8	5	
Lower extremity- Movement	Normal	41	81	0.015*
	Pathological	4	0	
Spine -Appearance	Normal	29	72	0.001*
	Pathological	16	9	
Spine- Movement	Normal	40	80	0.022*
	Pathological	5	1	
Gait -appearance	Normal	35	76	0.008*
	Pathological	10	5	
Gait- movement	Normal	40	81	0.005*
	Pathological	5	0	

*shows the presence of significant difference

Six patients (10%) had pes planus, which was the most common finding in patients. Other findings in patients in lower extremities were genu varum (1 patient), genu valgum (1 patient), genu recruvatum (2 patients) and increased knee flexion during gait (1 patient).

Concerning the spine and postural examination in CF patients, scoliosis in 10 patients (17%), increased thoracic kyphosis in 5 patients (5.1%), winged scapula in 3 patients (5.1%) and asymmetry in trapezius muscles in 6 patients (10.3%) were found. In sub-group analyses, there were no significant differences between genders considering the results of examination (*p* = 0.15).

The musculoskeletal pathologies in the control group were pes planus in 5 (5.3%), scoliosis in 3 (3%), increased thoracic kyphosis in 1 (1.02%), winged scapula in 1 (1.02%) and asymmetry in trapezius muscle in 4 (4%) of the participants. These pathologies also showed no significant differences among genders (*p* = 0.14) or did not show any correlations with age within the CF group.

### Evaluation of spine radiographs

Measurements of kyphosis and Cobb angles had high correlation between the two researchers (*r* = 0.99). Ten patients had scoliosis (17.2%), eight had increased thoracic kyphosis (13.7%). No vertebral fractures or other vertebral defects were detected on radiographs. When the diagnoses of scoliosis were compared between pGALS examination and radiograph measurements, there was a high level of correlation (*r* = 0.721 *p* = 0.001). There were no correlations among FEV1 and scoliosis or increased thoracic kyphosis. In sub-group analyses, the presence of scoliosis did not show a significant difference between males and females (*p* = 0.14).

### Postural stability parameters

In mCTSIB test, there were no significant differences between groups in sway velocity. Also, there were no significant differences between groups in parameters obtained in the walk across and STS test. There was an inverse correlation between the angle of thoracic kyphosis and weight transfer index of STS test (CC: −0.698, *p* = 0.01).

There was a significant decrease in the tandem walking speed in CF group while there were no differences in step width and sway velocity. Tandem speed correlated with FVC (*r* = 0.6 *p* = 0.03).

There were no significant differences between reaction times between the groups but reaction time negatively correlated with kyphosis angle (r:-0.398, *p* = 0.016).

According to the results of the LOS tests, CF group were significantly slower than the control group in going right and going left back (trials number 3 and 6).

About the EPE values, CF group has traveled significantly less in going forward and going forward-left (trials 1 and 7). There was a negative correlation between kyphosis angle and the EPE value of the 6th trial going to left backward. (r:-0.634, *p* = 0.04).

When CF and control groups were compared for directional control, there were significant differences in trials 1, 2 and 8, which are going forward, forward right and going back (Table 5).

**Table 5:** Significant differences in postural stability tests between groups summarized. Student’s t-test or Mann-Whitney-U test were used according to the distribution of the data

	CF group Mean ± Std Dev	Control group Mean ± Std Dev	*p* value
Tandem walking speed	20.47 ± 6.95	24.45 ± 7.79	0.030
MVL 3	5.73 ± 2.43	7.12 ± 4.88	0.030
MVL 6	4.90 ± 3.18	5.74 ± 2.52	0.050
EPE 1	63.22 ± 28.68	71.57 ± 25.73	0.026
EPE 7	83.89 ± 22.39	90.18 ± 26.89	0.027
DC 1	70.32 ± 29.39	78.01 ± 23.10	0.050
DC 2	67.62 ± 24.69	74.68 ± 18.58	0.050
DC 8	57.94 ± 31.30	70.41 ± 22.93	0.010

*MVL* Movement velocity, *EPE* end point excursion, *DC* directional control

There were no correlations between the presence of musculoskeletal abnormalities or the presence and severity of scoliosis and thoracic kyphosis with other LOS parameters.

There was no correlation between the vitamin D levels and BMI Z-scores of the patients and the postural stability parameters. In sub-group analyses, there were no differences between genders in any of the postural stability parameters.

## Discussion

This study shows that there is an increase in musculoskeletal pathologies in CF patients. There is also a slight involvement of postural stability in children with CF, albeit it is not a wide disparity as hypothesized in the beginning. Moreover, the influence of these musculoskeletal involvements in postural stability is present but the correlation is not strong.

In this study, pGALS was chosen as it is a systematic way of doing musculoskeletal examination in children [19]. It was also aimed to scan not only the spine but also the entirety of the musculoskeletal system to show a more detailed picture of musculoskeletal pathologies. Data was limited in the literature with children CF, with only a couple of studies that has examined the whole of musculoskeletal system [23, 24]. The suggested reporting method and the rapid way of applying the examination make it quite useful but may have overlooked some of the minor deficits. Even though, there are still significant differences in CF group and control group. One of the disadvantages of this method is that it may not be considered objective enough since it is a musculoskeletal examination without objective measurement tools or cut-off values. To decrease this discrepancy, spine radiographs were obtained and considered as the primary measurements in diagnosing scoliosis and increased thoracic kyphosis. There is a high correlation between the examination and radiographs, which shows that when in doubt, a physician should not deter from ordering a spine radiograph. An unexpected side benefit of using pGALS is that the scanning question ‘Do you experience difficulty going up and down the stairs?’ can differentiate normal children from patients with CF. It is shown in this study that even if there is mild involvement, exertional activities like taking the stairs are significantly affected in children with CF when compared to typically developing children.

This study has also shown a significant increase in the incidence of scoliosis and thoracic kyphosis in patients with CF concurrent with some of the previous research [2, 7, 8]. However, there is not a correlation between the presence of scoliosis or the increase in thoracic kyphosis and lung function in this study. A recent review investigating this relationship between posture and lung functions in patients with chronic obstructive diseases also resulted that the extent of alterations and its clinical impact is variable in these patients. The reason may be the lack of standard postural evaluations in the literature [25]. The other musculoskeletal pathologies have not been described previously. Most common pathology defined in this study is pes planus. Possible reasons for pes planus or other additional musculoskeletal pathologies are still not clear and beyond the scope of this study.

This study has documented that in assessments of postural stability, there is involvement in parameters of tandem walk speed and various parameters of limits of stability. Both are a part of dynamic stability, which seems to be affected more prominently in patients with CF. The direction of the movement that is affected is not consistent. Rather various parameters in different directions are affected. The device measuring postural stability is chosen based on its objectivity and its ability to detect minor changes in postural stability. It is known that patients with CF have impaired skeletal muscle oxidative capacity and this reduced skeletal muscle oxidative capacity contributes to exercise intolerance in patients with cystic fibrosis [26]. The involvement of dynamic stability can also be the result of decreased muscle oxidative capacity in patients with CF and can explain the fact that various parameters are involved in various tests. Therefore, future studies that can correlate these involvements or show an intervention increasing muscle oxidative capacity can improve postural stability would be of value. At this moment however, the exact clinical meaning of the involvement of postural stability pattern is obscure. The angle of thoracic kyphosis seems to be correlated with various postural stability parameters and influence postural stability in patients with CF. No other musculoskeletal pathology, neither the Cobb angle correlated with postural stability.

The extent of the pulmonary involvement of the patients and does not seem to contribute to the postural stability parameters. The only correlation found between lung capacities and postural stability parameters is the correlation between FVC and tandem walk speed. It is possible that functional capacity influences the speed of movement rather than other postural stabilities, but the exact clinical influence remains obscured in this setting.

The strengths of this study are the inclusion of all the patients above the age of 6, giving a wider range of patients, as opposing to the previous studies of similar nature that investigate mainly adults. Also, this study is about the full musculoskeletal examination of the patients with CF, not the spine alone. The use of an objective measure for postural stability is a plus, but to find more reliable results; patient pool needs to be expanded. As a limitation of this study, only 7 of the patients involved in this study had a severe involvement (FEV1 level below 40%). The obligation of involving pre-pubertal and milder patients, so that postural stability tasks could be performed, narrowed the patient pool. This may have caused weaker correlations and has been an essential drawback of the studies similar to this one before, which leads to a selection bias and type II error [27]. Furthermore, the pediatric pulmonology clinic that provides the patient pool for our study consists mainly of younger patients with only approximately 25% above the age of six. In Turkey, the awareness for cystic fibrosis has significantly increased in the last decade and it has been added to the newborn screening program as recent as 2015. Increased awareness will shift and expand the patient pool. Genetic analyses for CF have also been available in Turkey very recently. Patient pool of this study did not usually had a genetic diagnosis, and a new one could not be obtained due to lack of funding and the limitations applied by the ethical committee. It is becoming more widely available in the future and hopefuly will help understand the relations of genetics with the musculoskeletal involvement. Another limitation of this study is that the association of musculoskeletal impairments and other clinical pictures that can occur in CF (i.e. the presence of diabetes) is not investigated. In the future, another design, which can involve more patients especially with more severe involvement, and investigating the relation of a full clinical picture with musculoskeletal involvement, may answer more questions on this subject.

## Conclusions

In patients with CF, a simple but systematic musculoskeletal examination can detect lower extremity and spine pathologies, which are more frequent than the normal population. They influence postural stability to a certain level. However, exact causal relations need further investigation to be determined.
